# TGF-β/Smad Signaling Pathway in Tubulointerstitial Fibrosis

**DOI:** 10.3389/fphar.2022.860588

**Published:** 2022-03-24

**Authors:** Xiao-Yong Yu, Qian Sun, Ya-Mei Zhang, Liang Zou, Ying-Yong Zhao

**Affiliations:** ^1^ Department of Nephrology, Shaanxi Traditional Chinese Medicine Hospital, Xi’an, China; ^2^ Key Disciplines of Clinical Pharmacy, Clinical Genetics Laboratory, Affiliated Hospital and Clinical Medical College of Chengdu University, Chengdu, China; ^3^ School of Food and Bioengineering, Chengdu University, Chengdu, China

**Keywords:** chronic kidney disease, renal fibrosis, TGF-β/Smad signaling pathway, tubulointerstitial fibrosis, natural products

## Abstract

Chronic kidney disease (CKD) was a major public health problem worldwide. Renal fibrosis, especially tubulointerstitial fibrosis, is final manifestation of CKD. Many studies have demonstrated that TGF-β/Smad signaling pathway plays a crucial role in renal fibrosis. Therefore, targeted inhibition of TGF-β/Smad signaling pathway can be used as a potential therapeutic measure for tubulointerstitial fibrosis. At present, a variety of targeting TGF-β1 and its downstream Smad proteins have attracted attention. Natural products used as potential therapeutic strategies for tubulointerstitial fibrosis have the characteristics of acting on multiple targets by multiple components and few side effects. With the continuous research and technique development, more and more molecular mechanisms of natural products have been revealed, and there are many natural products that inhibited tubulointerstitial fibrosis *via* TGF-β/Smad signaling pathway. This review summarized the role of TGF-β/Smad signaling pathway in tubulointerstitial fibrosis and natural products against tubulointerstitial fibrosis by targeting TGF-β/Smad signaling pathway. Additionally, many challenges and opportunities are presented for inhibiting renal fibrosis in the future.

## 1 Introduction

Chronic kidney disease (CKD) has been recognized as a major public health problem worldwide and the global increase in CKD is associated with the increase in the prevalence of diabetes, hypertension, obesity, and aging (Webster et al., 2017; Hansrivijit et al., 2021). Recent data showed that global prevalence of CKD was estimated to be between 11% and 13% in 2016 (Hill et al., 2016). This number is continuous increase by 2030/2050 (Mantovani and Chiara, 2020). Moreover, with the progression of CKD, it can easily lead to the occurrence of cardiovascular diseases (Ravid et al., 2021). Renal fibrosis, characterized by tubulointerstitial fibrosis and glomerulosclerosis, is the common pathway and inevitable outcome of various progression of CKD to end-stage renal disease (ESRD) and need renal replacement treatments such as dialysis and transplantation (Humphreys, 2018; Aydin et al., 2019; Jain et al., 2019; Carta et al., 2020; Sawhney and Gill, 2020). Regardless of the underlying diseases, it has been demonstrated that patient’s survival is associated with tubulointerstitial fibrosis (Tampe and Zeisberg, 2014) and the extent of tubulointerstitial fibrosis on renal biopsy is thought as a prognostic indicator for CKD treatment (Tang et al., 2019). Fibrosis is a pathological scar formation process (Hu et al., 2020; Miao et al., 2021a). The histopathological characteristics of tubulointerstitial fibrosis are excessive synthesis and deposition of interstitial extracellular matrix (ECM) components, which are association with inflammatory cell infiltration, damage of tubular epithelial cells, activation and proliferation of fibroblasts and rarefaction of the peritubular microvasculature (Ma S.-X. et al., 2018; Humphreys, 2018; Bhargava et al., 2021; Medina Rangel et al., 2021). Renal fibrosis is associated with the activation of transforming growth factor-β (TGF-β)/Smad, Wnt/β-catenin, IκB/NF-κB and Keap1/Nrf2 signaling pathways (Chen DQ. et al., 2017; Chen et al., 2018a), the overexpression of renin-angiotensin system and aryl hydrocarbon receptor (Liu J.-R. et al., 2020; Miao et al., 2020; Miao et al., 2021b) and the dysregulation of metabolites such as amino acids and lipids (Chen et al., 2016a; Chen et al., 2019a; Feng Y. L. et al., 2019; Wang et al., 2021a). The robust pathological markers of CKD progression are the impairment of renal excretion function and the degree of fibrosis or fibroblast number, which are closely related to tubulointerstitial fibrosis (Chen et al., 2019b). The clinical first-line therapy for CKD is using anti-renin-angiotensin system remedies, such as angiotensin-converting enzyme inhibitors or angiotensin II type 1 receptor blockers. These therapies were proven to be beneficial for patients with CKD, protected against proteinuria and renal function decline, and ameliorated the progression to ESRD. However, considerable numbers of patients were still not being adequately treated and often progress toward ESRD. Therefore, it is urgent to find effective drugs to retard tubulointerstitial fibrosis and delay the progression of CKD. However, it is disappointing that no effective and satisfactory therapies in clinic settings have been found until now (Xu BH. et al., 2020).

Members of TGF-β superfamily have a common dimeric structure, which consist of six conserved cysteine residues, including bone morphogenetic protein, growth and differentiation factors, anti-Müllerian hormone, activin, inhibin, nodal and TGF-β (Chen and Ten Dijke, 2016; Hu et al., 2018). Among these effects, TGF-β is a key profibrotic mediator in fibrotic diseases (Hu et al., 2018). TGF-β is a multifunctional regulatory cytokine, which is involved in regulating a broad range of cellular processes, such as proliferation, differentiation, apoptosis, adhesion, migration, wound repair, and the pathogenesis of fibrosis (Meng et al., 2016a). Numerous studies have demonstrated that TGF-β/Smad pathway played a crucial role in renal fibrosis (Meng et al., 2016b; Hu et al., 2018). TGF-β is the major driver of fibrosis in CKD (Meng et al., 2016a) and also exerts protective effects by suppressing inflammation and inducing autophagy (Sureshbabu et al., 2016). Overexpression of TGF-β1 can elicit renal fibrosis by activating both canonica TGF-β/Smad signaling pathways and non-canonical signaling pathways including mitogen-activated protein kinase, p38, Rho-GTPases, phosphatidylinositol-3 kinase, Rac, Cdc42 and integrin linked kinase (Chen et al., 2018b), which results in activation of myofibroblasts, excessive ECM components and ECM degradation inhibition. In TGF-β/Smad signaling pathway, Smad proteins have a complex mechanism, which have competitive effects of profibrosis and antifibrosis. Furthermore, there are complex interactions between TGF-β/Smad signaling pathway and other signaling pathways (Meng et al., 2016b). Therefore, fibrotic mechanism is so complex that it is necessary to further understand the pathogenesis of renal fibrosis regulated by TGF-β/Smad pathway and find possible therapy.

Numerous publications have shown that TGF-β/Smad pathway played a key role in CKD caused by diverse etiologies, such as diabetic nephropathy (Li et al., 2020), hypertensive nephropathy (Liu et al., 2017), IgA nephropathy (Wang P. et al., 2018), post-transplant nephropathy (Wang Z. et al., 2018) and aristolochic acid nephropathy (Song et al., 2019). Moreover, with the gradual loss of renal function, these diseases will inevitably mediate the progression of renal fibrosis. The inhibition of TGF-β1/Smad signaling pathway retarded renal fibrosis in CKD (Meng et al., 2016a). Therefore, targeting TGF-β/Smad pathway against tubulointerstitial fibrosis is a promising therapeutic strategy. Increasing evidence has shown that natural products have been demonstrated to be as a potential therapy in a variety of disease (Chen et al., 2018a; Newman and Cragg, 2020). A number of natural products have been demonstrated to protect against tubulointerstitial fibrosis (Chen et al., 2018a). In this review, we summarized the effect of TGF-β/Smad signaling pathway on the tubulointerstitial fibrosis and highlighted the therapeutic potential of natural products by targeting TGF-β/Smad pathway against tubulointerstitial fibrosis.

## 2 TGF-β/Smad Signaling Pathway

**FIGURE 1:**
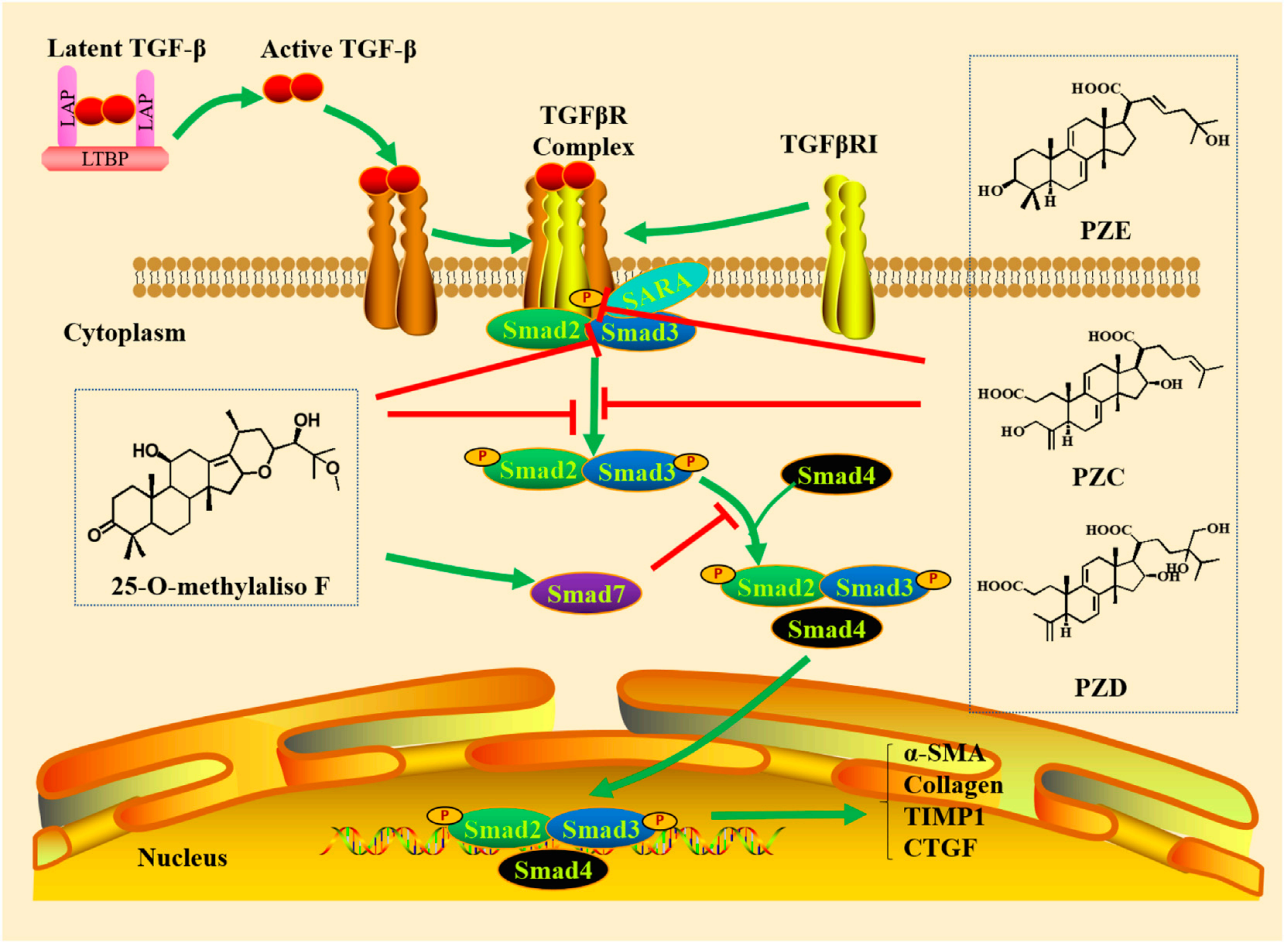
Canonical TGF-β/Smad pathway. TGF-β synthesized in precursor form combines with latency-associated peptide (LAP) that promotes attachment with LTBP through the formation of disulfide bonds, which is released from the LAP and LTBP and becomes active when exposed to various stimuli. The active TGF-β binds to TGFβRII, which recruits and activates TGFβRI through phosphorylated the GS domain in TGFβRI. TGFβRI phosphorylates and activates Smad2 and Smad3 (R-Smads). Smad4 combined with phosphorylated R-Smads to form a trimer complex for transcriptional regulation. Smad7 is involved and acts as a negative feedback regulation loop to regulate the target genes that regulate cell homeostasis.

It is well known that TGF-β/Smad signaling pathway is mainly composed of TGF-β and its receptors, as well as various Smad proteins. TGF-β1, TGF-β2 and TGF-β3 are three isoforms of TGF-β identified in mammals, which have 70-82% amino acid homology and induce different biological reactions (Chen H. et al., 2018; Ma and Meng, 2019). Among them, TGF-β1 can be produced by all types of resident kidney cells and is the most abundant and active isoforms (Meng et al., 2015). TGF-β combines with latency-associated peptide (LAP) that promotes attachment with latent TGF-β binding protein (LTBP) through the formation of disulfide bonds, which is released from LAP and LTBP and becomes active when received various stimulations, such as reactive oxygen species (ROS), integrins, proteases and metalloproteinases (Meng et al., 2013; Meng et al., 2015; Ma and Meng, 2019). TGF-β initiates intracellular signaling by binding to receptor complexes (Chen H. et al., 2018). TGF-β receptors include TGF-β receptors type I (TGFβRI), TGF-β receptors type II (TGFβRII) and TGF-β receptors type III (TGFβRIII). TGF-β1 and TGF-β3 directly bind to TGFβRII, while TGF-β2 needs TGFβRIII to bind to TGFβRII (Yu et al., 2003). Therefore, TGFβRII has high affinity for TGF-β ligands, and TGFβRI does not bind to TGF-β directly. After TGFβRII binds to TGF-β, TGFβRII recruits and activates TGFβRI through phosphorylated the GS domain in TGFβRI (Nakerakanti and Trojanowska, 2012). The TGFβRI and TGFβRII contain serine-threonine protein kinases in their intracellular domains, which can activate intracellular signaling by phosphorylated Smad protein (Blobe et al., 2000). TGFβRIII is the most abundant type, which increases the bioavailability of TGF-β to signal TGF-β receptor by binding TGF-β and then transferring it to its signaling receptors (Blobe et al., 2000; Nakerakanti and Trojanowska, 2012). The Smad family of human includes eight members (Smads 1–8), which are called receptor-regulated Smads (R-Smads). Generally, TGFβRI can phosphorylate and activate Smad2 and Smad3 in the TGF-β/Smad pathway. Smad4, called common mediator Smad (Co-Smad), is a common partner of all of the receptor-activated Smads. It is a specific transcription co-regulatory factor, which can be recruited to Smad transcription complex. Therefore, the trimeric complex formed by phosphorylated R-Smads and Smad4 can be used for transcriptional regulation. Smad6 and Smad7 are called inhibitory Smads (I-Smads), which can interplay with activated receptors and R-Smads to inhibit their activities. Smad7 is mainly involved and acts as a negative feedback regulation loop to regulate the target genes that regulate cell homeostasis (Macias et al., 2015). Therefore, TGF-β and various Smad proteins can interplay each other and work together to regulate various physiological and pathological mechanisms (Figure 1).

## 3 Role of TGF-β/Smad Signaling Pathway in Tubulointerstitial Fibrosis

### 3.1 Role of TGF-β in Tubulointerstitial Fibrosis

TGF-β is closely related ligands that have pleiotropic activity on most cell types of the body and play an important role in maintaining homeostasis in normal tissues. TGF-β1 is a major or predominant isoform involved in fibrosis and the effects of TGF-β2 and TGF-β3 may be partially mediated by TGF-β1. The overexpression of TGF-β1 is causally related to the progression of renal fibrosis caused by aging, diabetes, hypertension, obstruction, ischemia and toxin-induced injury (Gifford et al., 2021; Ma et al., 2022). TGF-β1 upregulation has been confirmed in patients and animal models with CKD (Hu et al., 2018). In addition, urinary TGF-β1 level, which reflects the degree of renal pathology and the development of CKD (Lawson et al., 2016), is positive correlated with renal fibrosis (Meng et al., 2015) and can be used as a clinical marker of renal fibrosis. Targeted TGF-β1 gene leads to multifocal and mixed inflammatory cell reaction in mice and conditional deletion of TGFβRII leads to kidney inflammation in mice (Meng et al., 2012a), which indicate that TGF-β1 is an effective anti-inflammatory molecule. However, blocking TGF-β1 signaling could eliminate its profibrotic effect, but inhibit its anti-inflammatory effect and thereby promote renal fibrosis (Sureshbabu et al., 2016). In addition, TGF-β1 could induce autophagy, promoting collagen degradation and cell protection for renal cell survival to prevent and treat renal fibrosis (Sureshbabu et al., 2016). Therefore, TGF-β is closely related to the formation of tubulointerstitial fibrosis and targeted inhibition of TGF-β overexpression is an important strategy for the treatment of tubulointerstitial fibrosis.

### 3.2 Role of Smad Proteins in Tubulointerstitial Fibrosis

#### 3.2.1 Role of Smad2 and Smad3 Proteins in Tubulointerstitial Fibrosis

It is well known that Smad2 and Smad3 are two important downstream genes of canonical TGF-β signaling that are activated in fibrotic kidneys (Jiang et al., 2019; Li et al., 2019). It is now well documented that Smad3 and Smad3-dependent non-coding RNAs are activated by TGF-β1 to transcriptionally and differentially regulate renal inflammation and fibrosis (Gu et al., 2020). The expression levels of TGF-β1 and Smad3 are elevated and pathological staining results show obvious fibrosis of renal tissues in rats with unilateral ureteral obstruction (UUO) (Tian et al., 2021). However, mice lacking Smad3 expression protected against renal fibrosis following UUO by blocking epithelial-mesenchymal transition (EMT) and eliminating monocyte influx and collagen deposition, and the cultured primary renal tubular epithelial cells from mice lacking Smad3 also revealed that Smad3 is critical for TGF-β1-mediated EMT. In addition, TGF-β/Smad3 pathway could be activated in patients with type 2 diabetic nephropathy. It was found that Smad3 wild-type db/db and Smad3^+/−^ db/db mice suffered from diabetic kidney injury, such as the decrease of renal function, a marked deposition of collagen I and collagen IV, and an increased macrophages in kidney. However, there was no evidence between renal fibrosis and inflammation response in Smad3 knockout db/db mice (Xu M. et al., 2020). Smad3 can also promote renal fibrosis by incorporating collagen promoter region and inhibiting ECM degradation *via* the induction of tissue inhibitor of matrix metalloproteinases (TIMP) while increasing the activity of matrix metalloproteinase-1 (MMP-1) (Gu et al., 2020). These facts indicate that Smad3 is the core of the pathogenesis of tubulointerstitial fibrosis (Sato et al., 2003). By contrast, Smad2 has renal protective effect. Despite the embryonic lethality of the Smad2 gene knockout mice (Gu et al., 2020), Smad2 conditional deletion from renal tubular epithelial cells and fibroblasts has been reported to significantly enhance renal fibrosis by increasing the expression of collagen I, collagen III, and TIMP-1 as well as decreasing the expression of MMP-2 to TGF-β1, which are due to the fact that Smad2 deletion promotes Smad3 phosphorylation, promoter activity, Smad3 binding to collagen promoter (COL1A2) (Meng et al., 2010). Therefore, targeted regulation of Smad3 expression is a potential target for treatment of tubulointerstitial fibrosis.

#### 3.2.2 Role of Smad4 Protein in Tubulointerstitial Fibrosis

Smad4 is a common Smad protein, which forms heteromeric complex with Smad2/3 and translocates into nucleus to mediate expression of downstream target genes (Meng et al., 2012b). It has been found that Smad4 could enhance Smad3-induced renal fibrosis by transcription and inhibit NF-κB-induced renal inflammation by Smad7-dependent mechanism, thereby exerting multiple effects (Lan, 2011). In streptozotocin-treated rats, the increased expression of TGF-β1 and Smad4 may lead to transcriptional regulation of target genes, which contributes to the progression of renal fibrosis (Yang et al., 2007). It is reported that siRNA Smad4 can inhibit renal fibrosis and α-smooth muscular actin (α-SMA)-positive myofibroblasts in mice induced by single intraperitoneal injection of folic acid (Morishita et al., 2014). The mechanism of inhibiting fibrosis of which may be explained by the fact that Smad4 deletion affects Smad3-mediated promoter activity and the combination of Smad3 and COL1A2 promoter, but does not affect p-Smad3 and nuclear translocation (Meng et al., 2016b). Furthermore, the destruction of Smad4 represses Smad7 transcription, thereby inhibiting NF-κB activation and promoting kidney inflammation as evidenced by inflammatory cells infiltration, upregulation of interleukin-1β (IL-1β), tumor necrosis factor-α (TNF-α), monocyte chemoattratctant protein-1 (MCP-1), and intercellular adhesion molecule-1 (ICAM-1) in conditional Smad4 knockout UUO mice (Meng et al., 2016a). Therefore, Smad4 has an important effect on renal fibrosis (Gu et al., 2020). The double action mechanism of Smad4 on kidney determines that it may not be an important anti-fibrosis target.

#### 3.2.3 Role of Smad7 Protein in Tubulointerstitial Fibrosis

Smad7 is a negative regulator and plays a negative role in renal fibrosis and inflammation (Meng et al., 2015; Gu et al., 2020). It is found that UUO model mice with Smad7 gene destruction can exhibit TGF-β/Smad2/3-dependent severe tubulointerstitial fibrosis by upregulating intrarenal TGF-β1 and connective tissue growth factor (CTGF) and activating Smad2/3, and sustain NF-κB activation, thereby enhancing intrarenal inflammation, including macrophage infiltration and expression upregulation of MCP-1, TNF-α, osteopontin and ICAM-1. The immunoreactivity for p-Smad2 and p-Smad3 are gradually increased in renal fibrosis, and Smad7 degradation and ubiquitination activities are significantly increased in UUO kidneys in mice, which indicate that decreased Smad7 protein caused by enhanced ubiquitin-dependent degradation leads to a pathogenic effect on renal fibrosis (Fukasawa et al., 2004). In streptozotocin-induced rats, insulin can upregulate Smad7 protein expression in the kidneys of diabetic rats, reduce ECM deposition, and delay the progress of diabetic nephropathy after controlling blood glucose, which may be related to the decrease of TGF-β1 and Smurf2 expression and attenuation of Smad7 ubiquitination in renal tissue (Wang YZ. et al., 2013). Therefore, during the process of fibrosis formation, Smad3 is generally activated whereas Smad7 is degraded by ubiquitin-proteasome degradation mechanisms. The imbalance between Smad3 and Smad7 is an key mechanism in renal fibrosis, which causes activation and accumulation of myofibroblasts, the excessive production of ECM and the reduction of ECM degradation (Meng et al., 2015). The latest study demonstrated that Latent TGF-β1 improved diabetic kidney disease through regulating Arkadia/Smad7 signaling (Wu et al., 2021). Therefore, targeting Smad3 inhibition and Smad7 promotion are important potential targets for treatment of tubulointerstitial fibrosis.

## 4 Crosstalk Between TGF-β1/Smad and Long Non-Coding RNA in Tubulointerstitial Fibrosis

**FIGURE 2:**
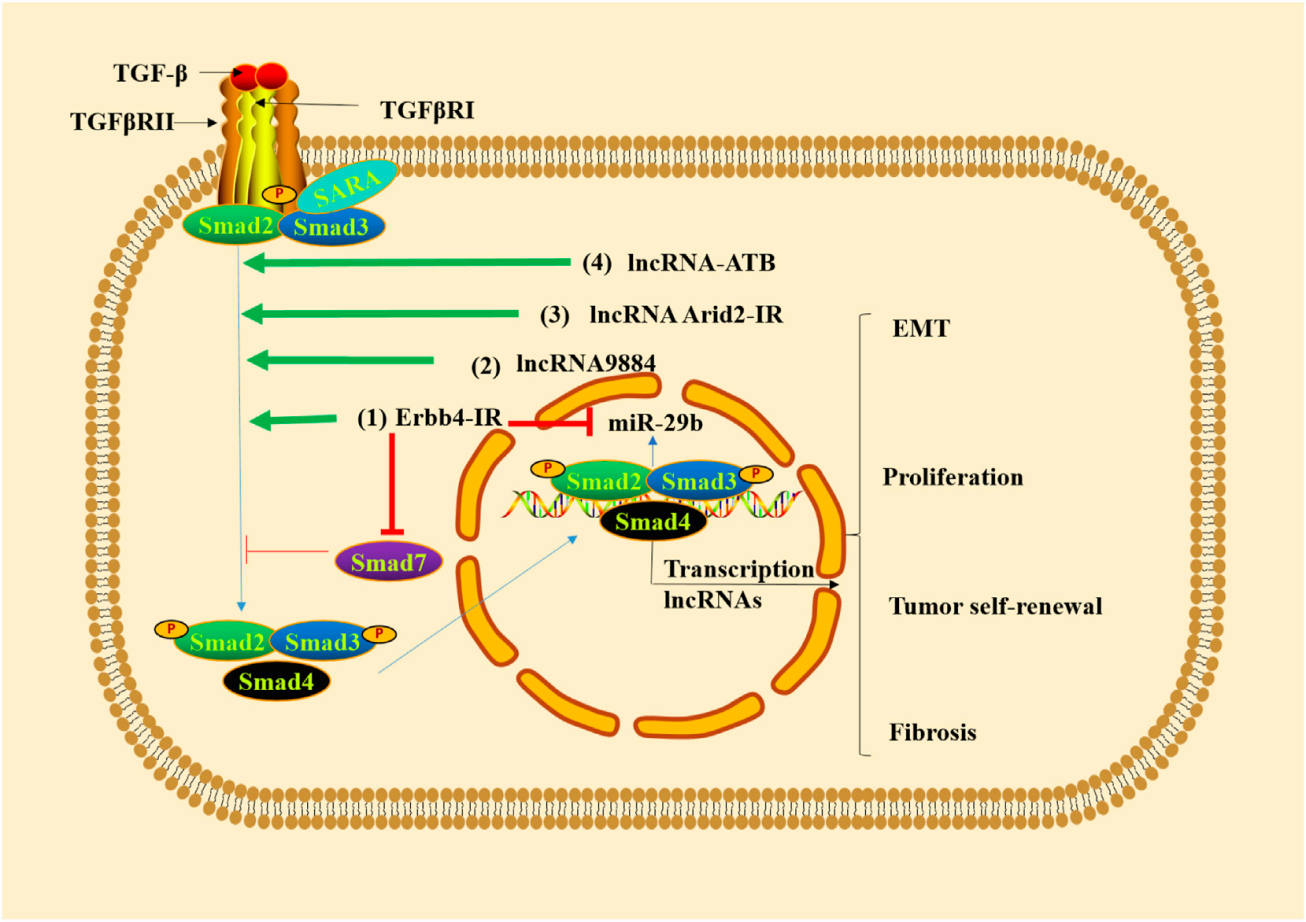
TGF-β1/Smad3 signaling regulates lncRNAs in renal fibrosis. (1) Erbb4-IR promotes renal fibrosis by suppressing miR-29b and downregulating Smad7. (2) lncRNA9884 is significantly upregulated in renal fibrosis. (3) The increased lncRNA Arid2-IR leads to ECM production in renal fibrosis. (4) lncRNA-ATB activates TGF-β/Smad2/3 pathway, thus promoting inflammation, cell apoptosis and senescence.

Long non-coding RNAs (lncRNAs) are an emerging class of non-coding RNAs, which are more than 200 nucleotides in length (Luo et al., 2020; Wang et al., 2021b). lncRNAs can regulate various biological functions at epigenetic, transcriptional and post-transcriptional levels (Huang, 2018) and bind DNA, RNA and protein (Wang Y.-N. et al., 2021). Mechanism studies have shown that TGF-β1/Smad3 is able to promote renal inflammation and fibrosis by regulating lncRNAs (Tang et al., 2017). Erbb4-IR is a Smad3-dependent lncRNA that promotes renal fibrosis by suppressing miR-29b of type 2 diabetic nephropathy in db/db mice (Sun X. et al., 2018) and is upregulated in the obstructed kidneys of UUO mice (Feng et al., 2018). Smad7 is a target gene of Erbb4-IR. The overexpression of Erbb4-IR can lead to renal fibrosis by downregulating Smad7 (Feng et al., 2018). The lncRNA9884 is a novel Smad3-dependent lncRNA, which is associated with diabetic renal injury. It can be significantly upregulated and enhance MCP-1-dependent renal inflammation in the diabetic kidney of db/db mice (Zhang YY. et al., 2019). lncRNA AT-rich interactive domain 2-IR (Arid2-IR) is identified as a Smad3-associated lncRNA in UUO kidney disease, the increasing of which can contribute to ECM production in diabetic kidney disease (Yang et al., 2019). Additionally, the overexpression of lncRNA-ATB can activate the TGF-β/Smad2/3 pathway, thus promoting inflammation, cell apoptosis and senescence in the TGF-β1-induced HK-2 cells, which indicates that silencing lncRNA-ATB is a new therapeutic strategy for intervention of renal fibrosis (Sun et al., 2020). All these evidence indicates that targeted regulation of lncRNAs involved in TGF-β/Smad pathway could improve tubulointerstitial fibrosis (Figure 2).

## 5 Natural Products as an Alternative Therapy Against Tubulointerstitial Fibrosis

### 5.1 The Isolated Compounds Against Tubulointerstitial Fibrosis

Traditional Chinese medicines (TCMs), a unique medical system with the remarkable feature of the use of multicomponent drugs, could hit multiple targets through multiple components (Chen et al., 2018a; Wang YN. et al., 2021). It pursues an overall therapeutic effect with a multi-ingredient treatment in the form of combination drug formulas in an attempt to improve therapeutic efficacy and lower drug side-effects and could also be an effective approach of reducing drug resistance (Jiang, 2005). Therefore, the studies of TCMs have attracted attention owing to its superiority in the treatment of refractory diseases (Chen et al., 2016b; Li et al., 2021a; Shao G. et al., 2021; Wang Y. W. et al., 2021; Yang and Wu, 2021). A variety of compounds isolated from natural products have been protected against renal fibrosis (Feng et al., 2019b; Luo et al., 2021; Shao M. et al., 2021; Zhao et al., 2011; Zhou F. et al., 2021). Therefore, natural products have attracted more and more attention around the world and are committed to the development of new drugs. With the continuous research and the development of technique, more and more molecular mechanisms of natural products have been revealed, and there are many natural products that inhibited tubulointerstitial fibrosis through TGF-β/Smad signaling pathway (Table 1) (Chen et al., 2018a; Hu et al., 2018).

**TABLE 1:** Summary of targets for natural products and their isolated compounds or extracts to exert anti-tubulointerstitial fibrosis effects through TGF-β/Smad signaling pathway.

Name	Compounds or extracts	Targets	References
*Radix astragali*	Astragalus injection	TGF-β1, TGFβRΙ, p-Smad3 and Smad7	Nie et al. (2014)
	Astragaloside IV	TGF-β1, p-Smad2/3 and Smad7	Wang et al. (2014)
	Astragalus polysaccharides	TGF-β1 and Smad3	Meng et al. (2020)
*Radix Salviae Miltiorrhizae*	Ethanol extracts	TGF-β, TGF-βRI, TGF-βRII, Smad2, Smad3 and Smad7	Cai et al. (2018)
	water extracts	TGF-β, TGF-βRI, TGF-βRII, Smad2 and Smad3	Cai et al. (2018)
	Salvianolic acid A	TGF-β1 and Smad3	Ma et al. (2016)
	Salvianolic acid B	TGF-β1, TGFβRII, p-Smad2 and Smad7	Tang et al. (2014); Zhang et al. (2019a)
	Protocatechualdehyde	Smad3 and Smad3-dependent lncRNA9884	Yang and Wu, (2021)
	Tanshinone IIA	p-Smad2/3	Tang et al. (2008)
*Poria cocos*	Poricoic acid ZA	p-Smad2/3	Li et al. (2017)
	Poricoic acid ZG and Poricoic acid ZH	p-Smad3	Wang et al. (2018a)
	Poricoic acid ZC, poricoic acid ZD and poricoic acid ZE	p-Smad3 and Smad3	Wang et al. (2018b)
	Poricoic acid A	Smad3	Chen et al. (2019e)
*Rhubarb*	*Rhubarb* extracts	TGF-β1, TGFβRI, TGFβRII, Smad2, p-Smad2, Smad3, p-Smad3, Smad4 and Smad7	Zhang et al. (2018)
	Chrysophanol	TGF-β1, p-Smad3, and Smad7	Dou et al. (2020)
	Emodin	TGF-β1 and Smad7	Ma et al. (2018a)
	Rhein	TGF-β1	Guo et al. (2001)
*Scutellaria baicalensis*	Baicalin	TGF-β1 and p-Smad2/3	(Hu et al., 2017; Zheng et al., 2017)
	Baicalein	TGF-β1 and p-Smad3	Hu et al. (2017)
	Wogonin	p-Smad3	Meng et al. (2016a)
*Alismatis rhizome*	25-O-methylalisol F	p-Smad3 and Smad7	Chen et al. (2018d)
*Tripterygium wilfordii*	Triptolide	TGF-β1, p-Smad2 and p-Smad3	(Cao et al., 2015; Sun et al., 2018a)
	Multi-glycoside	TGF-β1, Smad3 p-Smad2/3 and Smad7	Wan et al. (2014)
	Celastrol	Smad3	Tang et al. (2018)

*Radix Astragali* is used for treating kidney diseases (Zhang et al., 2014). Astragalus injection could mitigate diabetic nephropathy by increasing Smad7 expression, inhibiting the expression of TGFβRΙ, Smad3 and its phosphorylation as well as TGF-β1 mRNA levels (Nie et al., 2014). Astragaloside IV (AS-IV) could significantly mitigate tubulointerstitial fibrosis in the UUO rats and TGF-β1-stimulated NRK-49F cells. AS-IV could upregulate Smad7 expression, thereby blocking the upregulated protein expression of TGF-β1 and p-Smad2/3 (Wang et al., 2014). Astragalus polysaccharides could reduce the levels of creatinine and urea and inhibit collagen I, collagen III, collagen IV, α-SMA, TGF-β1 and Smad3 in rats treated by a high-fat diet with streptozotocin (Meng et al., 2020).

*Radix Salviae Miltiorrhizae* is usually used to treat cardiovascular diseases, but a number of studies have shown that it possessed protective effects on CKD (Cai et al., 2018). The ethanol and water extracts of *Radix Salviae Miltiorrhizae* prevented EMT through regulating NADPH oxidase/ROS/ERK and TGF-β/Smad signaling pathways in adenine-induced HK-2 rats (Cai et al., 2018). Several studies have demonstrated that salvianolic acid A and salvianolic acid B repressed renal fibrosis by modulating TGF-β1/Smad pathway. Salvianolic acid A reduced the expressions of TGF-β1 and Smad3 (Ma et al., 2016), while salvianolic acid B downregulated the expressions of TGF-β1 and p-Smad2 and preserved Smad7 expression (Zhang X. et al., 2019). Bioinformatics analysis revealed that TGFβRII might be a direct target of the miR-106b-25 cluster (miR-25, miR-93 and miR-106b). During the formation of EMT induced by TGF-β1, these miRNAs were found to be significantly downregulated, which were upregulated by salvianolic acid B treatment in a dose-dependent manner (Tang et al., 2014). This showed that salvianolic acid B could act on TGF-β receptor by targeting the specific miRNAs to retard renal fibrosis. Protocatechualdehyde, a phenolic acid, improved renal function and pathological changes *via* regulating Smad3 expression and NF-κB signaling in UUO-induced obstruction kidney. It also suppressed Smad3-dependent lncRNA9884 expression and inhibited inflammation by lncRNA9884/MCP-1 pathway (Yang et al., 2021). Tanshinone IIA, an active ingredient of *Radix Salviae Miltiorrhizae*, exerted its inhibitory effect on tubulointerstitial fibrosis by downregulating p-Smad2/3 expression in renal interstitial fibroblasts induced by TGF-β1 (Tang et al., 2008).

*Poria cocos* used in TCMs in China and Japan possessed the effects of diuretic, antiinflammation, antioxidative, antifibrosis and antihyperlipidemia (Wang Y. et al., 2013; Miao et al., 2015; Chen et al., 2016c). Earlier findings demonstrated the diuretic effect of the extracts of the surface layer of *Poria cocos* (Zhao et al., 2012; Feng et al., 2013). Further studies demonstrated that these extracts protected against tubulointerstitial fibrosis in adenine- and nephrectomized-induced chronic renal failure (Zhao et al., 2013a; Zhao et al., 2013b; Zhao et al., 2013c; Feng et al., 2019c). A number of poricoic acids, such as secolanostane tetracyclic triterpenoids and lanostane tetracyclic triterpenoids isolated and identified from *Poria cocos* protected against renal fibrosis by regulating Wnt1/β-catenin, IκB/NF-κB, and Keap1/Nrf2 pathways (Wang et al., 2017; Chen et al., 2019c; Wang et al., 2020). Poricoic acid A, a major component of *Poria cocos*, has been demonstrated to protect against renal fibrosis (Chen et al., 2019d; Chen et al., 2020a; Chen et al., 2020b). Previous study showed that poricoic acid A ameliorated AKI-to-CKD continuum by inhibiting Smad3 phosphorylation of renal tissues in renal ischemia-reperfusion injury rats (Chen et al., 2019e). The latest study demonstrated that poricoic acid A inhibited TGF-β1-induced renal fibrosis and proliferation through Smad3 and MAPK pathways (Li et al., 2021b).

New poricoic acid ZA protected against renal fibrosis through suppressing p-Smad2/3 through inhibiting Smad2/3-TGFβRI interaction (Li et al., 2017). Poricoic acid ZG and Poricoic acid ZH exerted their antifibrotic effects by selectively repressing the p-Smad3 *via* inhibiting the interactions of SARA with TGF-β1 and Smad3 (Wang et al., 2018a). Moreover, Poricoic acid ZC, poricoic acid ZD and poricoic acid ZE not only specific repressed p-Smad3 *via* blocking interplay between TGFβRI with Smad3, but also selectively suppressed Smad3 expression (Wang et al., 2018a). Structure-function analysis revealed that poricoic acid ZC and poricoic acid ZD, showed a strong inhibitory effect compared with poricoic acid ZE (Wang et al., 2018a). Therefore, compounds to the secolanostance skeleton showed strong bioactivity compared with lanostance skeleton.

*Rhubarb* is one of the most commonly used natural products, which contains a variety of chemical components that exhibit a variety of pharmacological activities, such as antitumor, antiinflammatory, lipid-lowing, hepatoprotective and renoprotective effects (Chen et al., 2015; Cao et al., 2017). Our earlier several publications showed that *Rhubarb* can ameliorate tubulointerstitial fibrosis in rat with adenine-induced CKD (Zhang et al., 2015; Zhang et al., 2016). Our studies show that *Rhubarb* extracts can suppress TGF-β/Smad3 signaling pathway by reducing the protein expressions of TGF-β1, TGFβRI, TGFβRII, Smad2, p-Smad2, Smad3, p-Smad3, and Smad4 and preserving expression of Smad7 protein (Zhang et al., 2018). Some studies have shown that chrysophanol could inhibit tubulointerstitial fibrosis by downregulating expression of TGF-β1 and p-Smad3 and preserving expression of Smad7 without affecting Smad2, Smad4 and TGF-β receptors in UUO mice (Dou et al., 2020). Mechanistically, chrysophanol repressed interaction between Smad3 and TGFβRI to suppress expression of p-Smad3 (Dou et al., 2020). These studies indicated that chrysophanol alleviated tubulointerstitial fibrosis by repressing Smad3 phosphorylation. In addition, emodin ameliorated renal fibrosis in rats *via* downregulating expression of TGF-β1 and Smurf2 and preserving Smad7 expression (Ma L. et al., 2018). Moreover, rhein inhibited cell hypertrophy and ECM accumulation mediated by TGF-β1 (Guo et al., 2001), which indicated that rhein exerted renoprotective effect, possibly by inhibiting the overexpression of TGF-β1. Rhubarb and astragalus capsule has been treated patients with CKD for decades. Recently, *in vivo* study showed that Rhubarb and astragalus capsule bunted tubulointerstitial fibrosis in the UUO rats by suppressing apoptosis *via* inhibiting TGF-β1/p38 MAPK pathway (Zeng et al., 2020). *In vitro* study demonstrated that drug-containing serum of rhubarb-astragalus capsule retarded EMT in the TGF-β1-induced HK-2 cells by downregulating TGF-β1/p38MAPK/Smad2/3 signaling axis (Qin et al., 2021).

*Scutellaria baicalensis* showed effects of cleaning away heat and dampness, fire and detoxification. Its extracts and compounds from *Scutellaria baicalensis* showed many pharmacological activities (Zhao et al., 2019). Baicalein showed a stronger inhibitory effect on cell proliferation, collagen synthesis, TGF-β1 and p-Smad3 upregulation than baicalin in the TGF-β1-induced NRK-49F cells (Hu et al., 2017). Furthermore, Baicalin has been demonstrated to decrease TGF-β1 level in serum and obstructed kidney, and repressed p-Smad2/3 in obstructed kidney of UUO rats (Zheng et al., 2017). In addition, wogonin inhibited upregulation of mRNA and protein of collagen I and α-SMA induced by TGF-β1 compared with wogonoside. Mechanistically, wogonin reduced p-Smad3 expression but showed weakened effect on non-canonical TGF-β pathway, such as p38, extracellular signal-regulated kinase 1/2 and mitogen-activated protein kinase pathways (Meng et al., 2016a).

*Alismatis rhizoma* exhibited lipid-lowing, diuretic and renoprotective activities (Tian et al., 2014; Miao et al., 2017). Our previous findings demonstrated the diuretic and anti-diuretic effects of *Alismatis rhizome* (Chen et al., 2014; Feng et al., 2014). Further studies demonstrated that these extracts protected against tubulointerstitial fibrosis in rats with adenine-induced CKD (Dou et al., 2018). Alisol B 23-acetate, as a major component of *Alismatis rhizoma*, ameliorated renal fibrosis by modulating renin–angiotensin system and gut microbiota–kidney signaling axis (Chen H. et al., 2020). Our previous study identified new compound 25-O-methylalisol F as a novel RAS inhibitor (Chen et al., 2018b). It could attenuate upregulated p-Smad3 and downregulated Smad7 without affecting the expression of phosphorylated Smad2, PI3K, ERK1/2 and p38 and expression of Smad4 in the TGF-β1- and angiotensin II-mediated NRK-52E and NRK-49F cells. It also specific repressed the combination of Smad3 with TGFβRI and Smad3 with SARA without affecting interaction of Smad2, TGFβRI and SARA (Chen et al., 2018b).

*Tripterygium wilfordii* is considered as an effective drug for treating diabetic nephropathy. Although it causes side effects, such as hepatic impairment, gastrointestinal reactions, menstrual disorders and reproductive effects, it is feasible to use it cautiously and appropriately to treat disease (Huang et al., 2020). Triptolide repressed proliferation, mediated apoptosis and led to cell cycle arrest in human mesangial cells (Sun SF. et al., 2018). Triptolide could upregulate Ski protein expression and downregulate protein expressions of α-SMA, TGF-β1 and p-Smad2/3 (Cao et al., 2015; Sun SF. et al., 2018). In addition, multi-glycoside of *Tripterygium wilfordii* could reduce ECM deposition and glomerulosclerosis by regulating mRNA or protein expressions of TGF-β1, Smad3, p-Smad2/3 and Smad7 in adriamycin-induced nephropathy rats (Wan et al., 2014). Moreover, celastrol could inhibit UUO- or TGF-β1-induced activation of Smad3, which can be candidate drug against renal fibrosis (Tang et al., 2018).

Compared with experimental researches, a number of studies have demonstrated that TCMs showed beneficial effects on patients with CKD (Chen et al., 2018a). Meta-analysis analysis showed that ligustrazine injection lowered the levels of creatinine and urea in serum as well as proteinuria, urine microalbumin and albumin excretion rate in patients with diabetic nephropathy including 858 treatment patients and 787 controls from 25 randomized controlled trial centers (Wang et al., 2012). In addition, ligustrazine blunted contrast-induced nephropathy in 148 patients with unstable angina (Ye et al., 2017). Recently, a randomized controlled trial showed Bupi yishen formula mitigated patients with non-diabetic stage 4 CKD (Mao et al., 2020). Moreover, a retrospective population-based cohort study showed that Chinese herbal medicine, such as *Salviae miltiorrhizae* and Jia-Wei-Xiao-Yao-San, mitigated CKD in patients with chronic hepatitis C (Chang et al., 2019). So far, although TCMs have been used for treating patients with CKD, few study demonstrated that TCMs were applied to treatment kidney diseases through targeting TGF-β/Smad signaling pathway.

### 5.2 The Traditional Chinese Medicinal Prescriptions Against Tubulointerstitial Fibrosis

There were a number of traditional Chinese medicinal prescriptions that inhibited renal fibrosis by regulating TGF-β/Smad pathway (Chen et al., 2018a; Yang et al., 2021). Oryeongsan (Wulingsan) is frequently used to treat nephrosis, dropsy and uremia. Oryeongsan could improve renal function, reduce mesangial expansion and downregulate expression of TGF-β1, Smad2, Smad4, Smad7 and collagen IV in the db/db mice (Yoon et al., 2014). You-gui Pill is treated to warm and recuperate “kidney-yang,” which could ameliorate tubulointerstitial fibrosis by downregulating translocation of p-Smad2/3 (Wang et al., 2015). Shenqiwan could ameliorate renal fibrosis by inhibiting mRNA and protein expressions of p-Smad2/3 *via* upregulating Smad7 in adenine-induced rats (Chen H. et al., 2017). HuangQi decoction inhibited protein expressions of α-SMA, collagen I, collagen III and collagen IV through downregulating the expressions of TGF-β1, TGFβRI, TGFβRII, Smad4, Smad2/3, p-Smad2/3 and preserved Smad7 protein expression in the UUO mice (Zhao et al., 2016). Fu-Fang-Jin-Qian-Cao granules, widely used to treat nephrolithiasis, alleviated renal EMT and fibrosis induced by calcium oxalate *via* downregulating protein expressions of TGF-β1, TGFβRI, TGFβRII, p-Smad3 and p-Smad2, and preserving Smad7 protein expressions (Liu W. et al., 2020). Bu-Shen-Jiang-Ya decoction can relieve renal fibrosis in Dahl salt-sensitive rats by inhibiting protein expressions of TGF-β1 and Smad2/3 (Liu WR. et al., 2020). Uremic Clearance Granules attenuated renal dysfunction and renal fibrosis by downregulating protein expressions of TGF-β1, TGFβRI, p-Smad2/3 and Smad4, and preserving Smad7 protein expression in the kidney of rats induced by adenine and UUO (Huang et al., 2014). Tangshen formula has been demonstrated to slow diabetic kidney disease in humans and animals. The latest findings indicated that Tangshen formula retarded renal fibrosis in rats with diabetic kidney disease by repressing TGF-β1/Smad3 signaling and lncRNA MEG3 expression (Zhou X. F. et al., 2021).

There are a variety of natural products and Chinese medicine compounds formed by different compatibility compositions of natural products. Further in-depth studies of the underlying mechanism of natural products through TGF-β/Smad signaling pathway is beneficial to the popularization and application of natural products.

## 6 Conclusion

The common pathway of progression of various CKD is renal fibrosis, so making every effort to prevent progression of renal fibrosis can effectively reduce global economic burden. In the early development of fibrosis, inhibiting or eliminating pathogenical factors and the use of antifibrotic agents can partially or completely inhibit renal fibrosis. However, due to the strong compensation of glomerular filtration function, the renal function may be unaffected or slightly damaged, so the patients often have no symptoms, thereby missing the optimal treatment opportunity. In view of the important role of TGF-β/Smad pathway in renal fibrosis, inhibition of renal fibrosis by targeting potential targets of this pathway will be an attractive therapeutic approach. TGF-β1 is considered to be the key mediator involved in fibrosis. Smad3 is the core of the pathogenesis of interstitial fibrosis. Smad2 and Smad7 have the renal protective effect. Smad4 enhances Smad3-mediated renal fibrosis by transcription and inhibits NF-κB-driven inflammation *via* a Smad7-dependent molecular mechanism. Therefore, targeting TGF-β1 and its receptors as well as its downstream Smad proteins and Smad-dependent lncRNAs is regarded as one of the feasible therapeutic strategies.

Currently, the renin-angiotensin system blockade is central to the treatment of patients with CKD, the renoprotective effects of which are aimed at preventing or slowing the progression of ESRD (Lee et al., 2015), but its efficacy is limited. Furthermore, the incidence of CKD increases year by year and has reached epidemic proportions (Wang et al., 2021a). It means that we will face great challenges to find more effective drugs for treatment of renal fibrosis. Meanwhile, natural products have unique advantages in inhibiting renal fibrosis and many natural products have been proved to target TGF-β/Smad signaling pathway to treat renal fibrosis, which means that we will have more opportunities to explore new treatments for renal fibrosis. Although there is a long way to go to suppress renal fibrosis in the future, we are confident that the more frustrated we are on this road, the more courageous we are, and we firmly believe that new treatments will always be found.

## Data Availability

The raw data supporting the conclusions of this article will be made available by the authors, without undue reservation.
